# Mathematical expertise modulates the architecture of dorsal and cortico-thalamic white matter tracts

**DOI:** 10.1038/s41598-019-43400-6

**Published:** 2019-05-02

**Authors:** Hyeon-Ae Jeon, Ulrike Kuhl, Angela D. Friederici

**Affiliations:** 1 Department of Brain and Cognitive Sciences, Daegu Gyeongbuk Institute of Science and Technology (DGIST), Daegu, 42988 Korea; 2 Partner Group of the Max Planck Institute for Human Cognitive and Brain Sciences at the Department for Brain and Cognitive Sciences, DGIST, Daegu, 42988 Korea; 3 Department of Neuropsychology, Max Planck Institute for Human Cognitive and Brain Sciences, Leipzig, 04103 Germany

**Keywords:** Cognitive control, Intelligence

## Abstract

To what extent are levels of cognitive expertise reflected in differential structural connectivity of the brain? We addressed this question by analyzing the white matter brain structure of experts (mathematicians) versus non-experts (non-mathematicians) using probabilistic tractography. Having mathematicians and non-mathematicians as participant groups enabled us to directly compare profiles of structural connectivity arising from individual levels of expertise in mathematics. Tracking from functional seed regions activated during the processing of complex arithmetic formulas revealed an involvement of various fiber bundles such the inferior fronto-occipital fascicle, arcuate fasciculus/superior longitudinal fasciculus (AF/SLF), cross-hemispheric connections of frontal lobe areas through the corpus callosum and cortico-subcortical connectivity via the bilateral thalamic radiation. With the aim of investigating expertise-dependent structural connectivity, the streamline density was correlated with the level of expertise, defined by automaticity of processing complex mathematics. The results showed that structural integrity of the AF/SLF was higher in individuals with higher automaticity, while stronger cortico-thalamic connectivity was associated with lower levels of automaticity. Therefore, we suggest that expertise in the domain of mathematics is reflected in plastic changes of the brain’s white matter structure, possibly reflecting a general principle of cognitive expertise.

## Introduction

Researchers have strived to reveal the underlying neural mechanisms of the mesmerizing performance exhibited by experts. Along with the development of neuroimaging techniques, a myriad of studies has shown expertise dependent modulation of functional patterns, including dynamic changes of task-dependent activation and functional connectivity across brain areas^1–8^. From a structural perspective, evidence suggests that becoming an expert modulates the brain’s architecture, inducing specific changes in grey matter volume^9–13^, cortical thickness^14,15^ and white matter structure^11,16–18^.

Understanding how such an expertise-dependent modulation in the brain—in terms of either function or structure—relates to inter-individual differences in behavior across experts and non-experts lies at the heart of studying respective neural correlates. Therefore, measuring expert behavior appropriately is critical to confirm that neural findings reflect a genuine effect of expertise^19^. Unfortunately, neuroimaging studies that focus on structural brain changes sometimes overlook the significance of associating behavior with brain data when attempting to characterize neural correlates of expertise, which causes three problems. Firstly, various studies compare pre-defined expert groups based on vocational qualification along with controls^10,15,18,20,21^. However, an occupation-based definition disregards individual variation within the expert and non-expert groups that may also be reflected in terms of neural differences. Secondly, in correlational studies, researchers have associated changes in brain structures with training time^22^, resting on the assumption that repeated practice leads to the development of expertise^23^. However, practice time explains only part of the variance in individual performance in complex tasks^24^. Thirdly, correlational analyses prominently describe structural changes in the brain related to speed-up of processing regardless of an improvement of performance^16,25^ or increases of performance independent of time needed to complete tasks^26^. However, one of the defining behavioral features of outstanding performance in experts is enhanced automaticity characterized as fast speed of processing while maintaining high levels of accuracy^27–30^. Therefore, previous investigations of brain plasticity in expertise do not seem to fully capture specific correlates of expert level processing, missing the critical link between neural correlates and automaticity of behavioral performance.

Enhanced automaticity is characterized as a reorganization (routinization) of serial execution of component processes with decreasing requirements for attention^28,31–33^. It shows that considerable amounts of information are organized and stored in the long-term memory of experts^34^. With practice, people gradually build up chunks of information to represent and process their knowledge with fewer steps^1^. Consequently, experts show effortless processing of relevant knowledge in their areas of expertise to which they have quick and reliable access^35,36^. As automaticity is a defining factor of expert performance, it will be an overarching attempt to interrogate neural dynamics of functions and structures in the brain in relation to automaticity in experts’ behavior.

Recently, using functional magnetic resonance imaging (fMRI), we investigated the functional specificity and connectivity in experts’ brain mediated by levels of automaticity in the processing of complex arithmetic formulas, with the aim of understanding the neural underpinnings of expertise linked to exceptional performance^3^. Experts functionally showed focal activation in the left precentral gyrus (PrCG) whereas non-mathematicians depicted a broad pattern of activation spanning the anterior-posterior axis of the prefrontal cortex (PFC). This result indicated that the level of mathematical expertise produced a modulating effect on the functional specification of the PFC. Moreover, this pattern was correlated with participants’ behavioral index of automaticity in mathematics, supporting a close relationship between automatized information processing and neural efficiency reflected by a decreased involvement of controlled or attentional processes primarily in frontal regions of experts^33,37^. We also observed expertise-dependent functional connectivity using psychophysiological interaction (PPI). Here, proficient participants recruited a fronto-parietal network whereas people with lower proficiency relied on fronto-striatal connections, supporting the divergent involvement of long-range connections determined by mathematical expertise.

While our previous work^3^ clearly demonstrated a modulatory effect of mathematical expertise with respect to functional specificity and connectivity in the human brain, the question for concomitant changes affecting anatomical structure remains unanswered. In accordance with the principle of neuroplasticity, that is, the assumption that experience constantly alters the brain’s structural organization^38^, anatomical differences between mathematicians and non-mathematicians are also to be expected. In fact, a comprehensive analysis of structural correlates of expertise might elucidate the dynamic changes in the brain when it comes to mathematicians’ outstanding performance. Most studies investigating structural connectivity in relation to mathematical processing have focused on numerical cognition in children, normal controls, or patients with dyscalculia (for a review, see Moeller *et al*.^39^). Expanding the research question to anatomical signatures of expertise will augment our knowledge of structural alterations induced by increased proficiency, thereby extending the established understanding of the roles of distinct white matter pathways within the scope of expertise.

**Figure 1:**
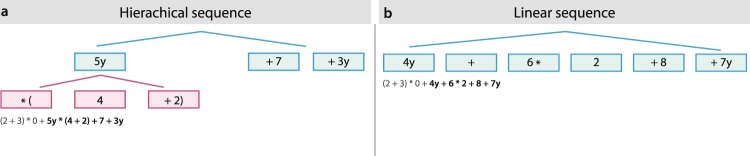
A schematic illustration of conditions in the previous fMRI experiment. (**a**) The complex arithmetic formulas comprise two parts. One is exemplified by “5y + 7 + 3y” (blue) having a long-distance computation between “5y” and “3y”. The other is exemplified by “(4 + 2)” (pink) being an inserted formula attached to “5y” using a multiplication symbol (*). (**b**) The simple arithmetic formulas only have the long-distance computation between “4y” and “7y” without an inserted formula. Here we have provided tree structures of algebraic expressions to help understanding of the formulas. In the actual experiment, the six stimuli were visually presented one by one (denoted by a square) after the lead-in stimulus “(2 + 3) * 0+”. Adapted, with permission, from^5^.

Therefore, in the present study we scrutinized the anatomical connections of an expert group (mathematicians) in comparison with normal controls (non-mathematicians) using diffusion-weighted magnetic resonance imaging (dMRI) that assesses the connectivity of white matter tracts between brain regions^40^. We evaluated structural connectivity in mathematicians and non-mathematicians using probabilistic tractography^40^, a method that enables us to draw indirect conclusions about specific functions of fiber tracts from the functional characteristics of their target regions^41^. The target areas of the present study, being used as regions of interests (ROIs) for the tractography, were located in the brain regions from our previous study^3^. Specifically, we selected all clusters exhibiting common activation for both mathematicians and non-mathematicians in the processing of complex arithmetic formulas (Fig. 1a compared to Fig. 1b). These areas comprise the left insula, left PrCG, left superior parietal lobe (SPL) and bilateral medial premotor cortex (mPMC) encompassing the anterior cingulate cortex (ACC). Since these regions were not only observed in our study but also in other studies where people were involved in numerical cognition or solving mental arithmetic formulas^4,39,42,43^, we chose these as the ROIs for our probabilistic tractography.

In particular, we investigated the contribution of the white matter tracts connecting these ROIs with other regions in the brain. Given their location, several adjoining tracts qualify as potential candidates supporting expertise-dependent processing. For instance the arcuate fasciculus/superior longitudinal fasciculus system (AF/SLF), connecting the temporal and frontal lobes via the parietal cortex^44^, has been scrutinized with respect to experts’ performance (e.g., chess players, phonetics experts or musicians)^45–47^ or training-related changes (e.g., reading, music or mathematics)^16,17,48^. Moreover, the AF/SLF has been known to support mathematics^42,49^, mathematics learning^50^ and mental arithmetic skills^51,52^. Along with the AF/SLF, cortico-thalamic connections also deserve attention, as they connect various cortical regions such as dorsolateral PFC, lateral orbital cortex, or ACC with the thalamus. These connections contribute to a wide range of cognitive processes that encompass learning, memory, inhibitory control and decision-making^53,54^. Previous studies provide evidence that the strength of the cortico-thalamic connection covaries with individual levels of mathematical proficiency as well as cognitive control^3,55,56^. Taken together, we expect an inextricable link between the involvement of the AF/SLF as a major cortico-cortical pathway along with the cortico-thalamic pathway and automatic processing in mathematical expertise.

The goal of the present study was to investigate to what extent structural connectivity measured by dMRI was modulated by the mathematical expertise, providing a comprehensive view of the neural mechanisms of expertise in mathematics. As mentioned above, we previously showed a distinct difference between mathematicians and non-mathematicians in their pattern of functional activation and connections^3^. In the present study, fiber tracking from seed ROIs that were commonly activated in both groups was conducted to assess structural integrity of the observed white matter structures in terms of their streamline density. Next, we examined the relationship between structural coherence of these tracts and mathematical expertise by correlating the streamline density with the coefficient of variation in reaction times (CV_RT_). CV_RT_ is known to provide an index of processing automaticity related to the level of expertise^28,31,32,36^. We hypothesized that fiber tracts connecting regions such as PrCG, insula, mPMC/ACC, and SPL would differentially support the processing of complex arithmetic formulas between experts and non-experts. Consequently, distinct correlations between streamline density of fiber tracts and CV_RT_ were expected. More specifically, we hypothesized that the AF/SLF would show a high streamline density as the level of mathematical automaticity increased, based on the well-known involvement of AF/SLF in experts’ performance^16,17,45,47,48,57^. On the contrary, we expected increased cortico-subcortical connectivity between the medial PMC (mPMC)/ACC and thalamus with decreasing levels of mathematical automaticity, based on the pivotal role of fronto-striatal connections during demanding cognitive processes^32,55,58–60^.

## Results

### Higher levels of automaticity in mathematicians compared with non-mathematicians

The CV_RT_ as an index of processing automaticity was calculated for both mathematicians and non-mathematicians while they computed arithmetic formulas. By running a Shapiro-Wilk normality test, we found that the CV_RT_ values were not normally distributed (W = 0.9418, p-value = 0.0481). Therefore, we performed a Wilcoxon ranked-sum test to assess group differences. Mathematicians showed a significantly higher degree of automaticity when processing complex arithmetic formulas in comparison with non-mathematicians (W = 360, p < 0.0001).

### Fiber tracts involved in the processing of complex arithmetic formulas across the groups

**Figure 2:**
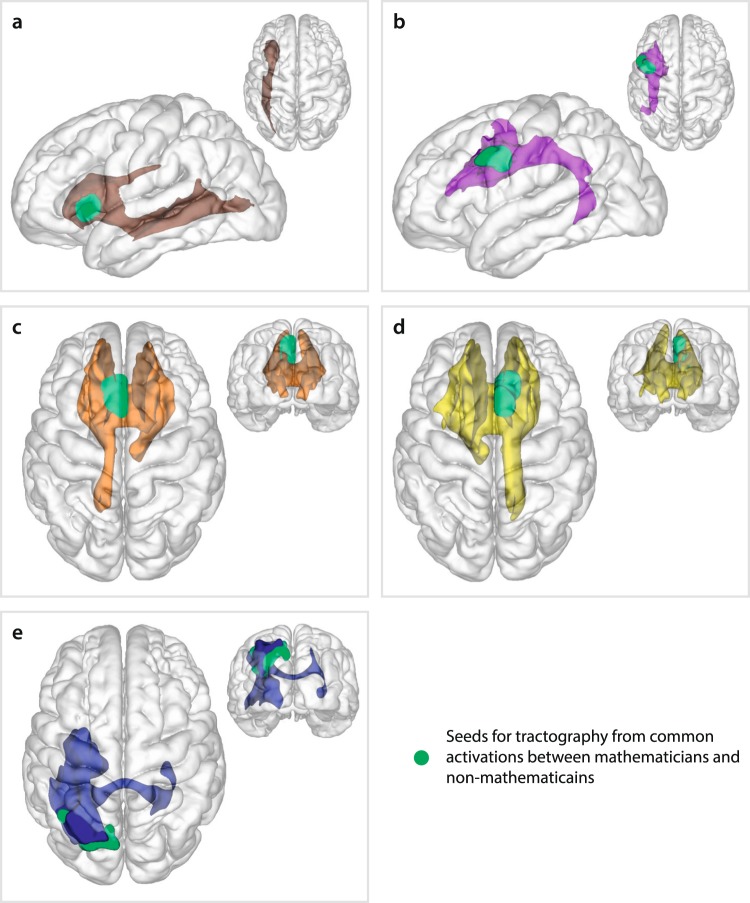
Tract masks at the group level in MNI space and corresponding seed regions. The masks were derived by averaging and thresholding the normalized tractograms aligned to the MNI152_T1_1mm_brain.nii.gz image as provided in FSL. Seed region of interests (green blob) are functional clusters which were commonly activated in complex arithmetic condition compared with simple arithmetic condition in the mathematics domain in the previous fMRI study^3^. (**a**) IFOF (brown) seeded in left insula, (**b**) dorsal pathway D1 (violet) seeded in the left PrCG, (**c**) corpus callosum, bilateral anterior thalamic radiation, and left cingulum (orange) seeded in left mPMC/ACC, (**d**) corpus callosum, bilateral anterior thalamic radiation, and right cingulum (yellow) seeded in right mPMC/ACC, (**e**) corpus callosum, IFOF, and corticospinal tract (navy) seeded in left SPL. (ACC, anterior cingulate cortex; IFOF, inferior fronto-occipital fascicle; PrCG, precentral gyrus; mPMC, medial premotor cortex; SPL, superior parietal lobule).

We initiated fiber tracking from seed ROIs that were commonly activated in both mathematicians and non-mathematicians^3^. Figure 2 shows average tract masks that served as basis for statistical analysis, generated from seeding within the respective ROIs. Seeding in the left insula yielded streamlines along the ventrally located inferior fronto-occipital fascicle (IFOF), which connects frontal regions with the posterior temporal and occipital cortex (Fig. 2a). The cluster in the left PrCG projected dorsally via the AF/SLF, connecting the PMC to STG and MTG (Fig. 2b). Seeding in the two medially located left and right mPMC/ACC clusters yielded cross-hemispheric connections of the frontal lobe areas through the corpus callosum, as well as cortico-subcortical connectivity via the bilateral thalamic radiations (Fig. 2c,d). Finally, seeding in the left SPL revealed cross-hemispheric projections along the corpus callosum as well as ventral connections as part of the IFOF and corticospinal tract (Fig. 2e).

### Cortico-cortical and cortico-thalamic pathways correlated differently with mathematical expertise

**Figure 3:**
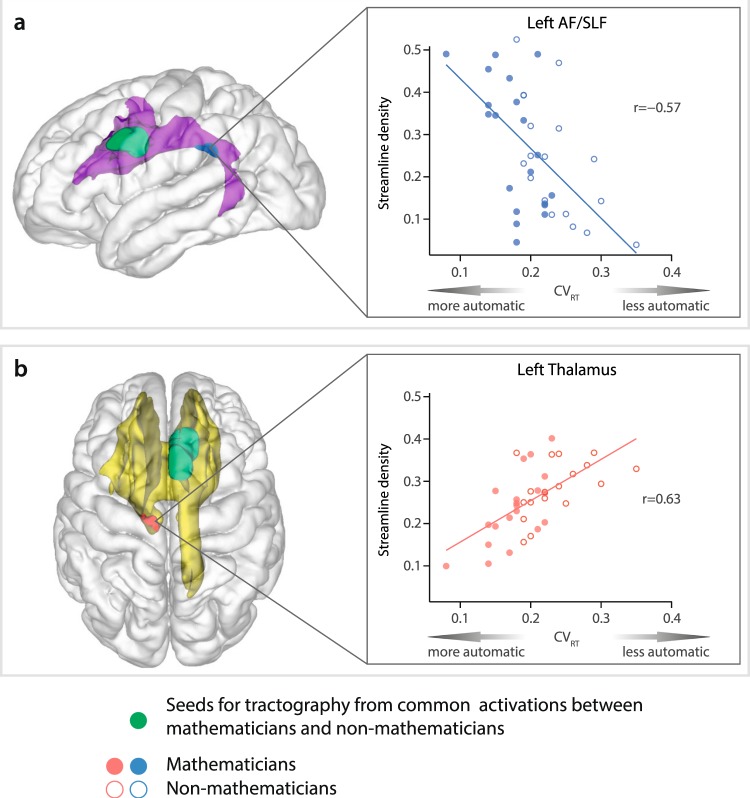
Clusters of significant negative and positive correlation between streamline density and CV_RT_ scores across mathematicians and non-mathematicians. (**a**) Seeding in the left PrCG (green) showed negative correlation between CV_RT_ and streamline density having its peak (blue) being located in the AF/SLF (violet). (**b**) Seeding in the right mPMC/ACC (green) yielded positive correlation between CV_RT_ and streamline density having its peak (red) being positioned, specifically in thalamus (a part of yellow tract). Reported clusters are size corrected at p < 0.05 and Bonferroni corrected for the number of seed regions. (AF/SLF, arcuate fasciculus/superior longitudinal fasciculus; ACC, anterior cingulate cortex; PrCG, precentral gyrus; mPMC, medial premotor cortex; SPL, superior parietal lobule).

We correlated streamline densities along the identified fiber bundles (Fig. 2) with the participant’s individual CV_RT_ scores to quantify which of the fiber tracts were specifically related to the level of mathematical expertise. We found two significant fiber tracts. Figure 3(a) shows a significant cluster (blue, r = −0.57, 67 voxels) denoting a negative correlation of streamline density with CV_RT_ residing within a cortico-cortical pathway, that is, the left AF/SLF pathway after seeding in the left PrCG. Thus, the relative number of streamlines within this cluster increased as the participant’s automaticity in the processing of mathematical formulas increased (denoted by decreased CV_RT_). Conversely, shown in Fig. 3(b), we found a positive correlation between CV_RT_ and the streamline density in a cortico-thalamic pathway (red, r = 0.63, 94 voxels) positioned within the left thalamus after seeding in the right PMC/ACC. Therefore, streamline density in this cluster increased as the participant’s automaticity in the processing of mathematical formulas decreased (indicated by increased CV_RT_) (Table 1).

**Table 1:** Fiber tracts showing a significant correlation with CV_RT_.

Seeds				Clusters correlated with CV_RT_					
Area	Center of gravity (x, y, z)			Area	Coordinates (x, y, z)			Voxel size	Correlation coefficient (r)
Left PrCG	−43	6	31	Left AF/SLF	−34	−39	25	67	−0.57
Right mPMC	8	23	38	Left thalamus	−7	−18	−3	94	0.63

Clusters are corrected at p < 0.05 and Bonferroni corrected for the number of seed regions. (AF/SLF, arcuate fasciculus/superior longitudinal fasciculus; mPMC, medial premotor cortex; PrCG, Precentral Gyrus;).

In order to make sure that the direction and significance of the observed associations were not driven by either mathematicians or non-mathematicians alone, we additionally performed correlation analyses of mean streamline density within the previously identified clusters and CV_RT_ scores for each group separately. These, too, became highly significant, replicating the direction of the respective correlation within the whole study sample. Specifically, for streamline density within the left AF/SFL cluster, there was a significant negative correlation with CV_RT_ scores for mathematicians (r = −0.59, p = 0.0063) and non-mathematicians (r = −0.63, p = 0.0053), respectively (see Supplementary Fig. S1). Likewise, the association between our structural measure within the left thalamus cluster and our automaticity score was also significant (mathematicians: r = 0.68, p = 0.0009; non-mathematicians: r = 0.49, p = 0.0391). An additional statistical comparison of both sets of correlations using Fisher’s z revealed no significant differences between the strength of associations between both groups (AF/SLF cluster: z = −0.1800, p = 0.8572; left thalamus cluster: z = −0.8273, p = 0.4081).

## Discussion

The present study clearly demonstrated that the architecture of cortico-cortical and cortico-thalamic white matter tracts is modulated by mathematical expertise, with a diverging involvement of AF/SLF and thalamic pathways. More specifically, we found that the streamline density within the AF/SLF and thalamic pathways was differentially correlated with the degree of automaticity in mathematical expertise. Participants exhibiting a high level of automaticity showed increased streamline density in the left AF/SLF, whereas the streamline density in the left thalamic pathway was decreased. The novelty of the present study is that we investigated the structural connectivity of white matter tracts associated with the level of automaticity in processing complex arithmetic formulas within experts (mathematicians) and non-experts (non-mathematicians).

### The dorsal pathway and mathematical processing

We found that the dorsal pathway consisting of the AF/SLF support mathematical processes. This finding regarding the functional interpretation should be considered with caution because the function of fiber tracts can only be interpreted indirectly by functional activation of the grey matter^61^. The AF/SLF is known to have anatomically distinguished sub-parts^62,63^. The detailed discussion of the sub-parts of the AF/SLF is beyond the scope of the present study. However, it is important to note the distinction of two sub-parts of a dorsal connections: a direct pathway (long segment) connecting from temporal cortex to Broca’s area, and an indirect pathway (anterior and posterior segments) connecting from temporal cortex to PrCG via the parietal cortex^62,63^. The parietal cortex, being a region comprising an integral proportion of the indirect pathway of AF/SLF, has been known to be a crucial area implicated in mathematical cognition including intraparietal sulcus, superior parietal lobule and angular gyrus^39,64,65^. Therefore, the involvement of AF/SLF in the present study may be adduced in support of arithmetic calculation.

Previous studies have investigated the structural connectivity in numerical cognition (for a review, see Moeller *et al*.^39^). For instance, difficult addition that requires bridging to ten (e.g., 28 + 47) or magnitude processing in healthy adults are processed by dorsal connections as part of the AF/SLF system along with ventral fiber connections, whereas ventral tracts such as the middle longitudinal fascicle were predominantly found supporting processing of easy addition problems (e.g., 28 + 41)^42^. Another study also supports this dissociation of dorsal and ventral connections with respect to task difficulty, showing that difficult numerical magnitude processing is specifically supported by the dorsal AF/SLF as well as ventral connectivity including the external/extreme capsule fiber system^66^. Considering the experimental design of our previous functional MRI study, the processing of complex arithmetic formulas was more difficult than mere numerical fact retrieval involved in simple single-digit addition or retrieval of multiplication table facts. Therefore, our observation of the AF/SLF conforms to the previous studies suggesting a supporting role of the dorsal tract in the processing of relatively complex formulas. In the next section, we discuss the AF/SLF pathway in relation to the degree of automaticity in mathematics in more detail.

### Streamline density of the dorsal pathway related to automatic processing in mathematics

It has been shown that the speed of information processing is dependent on several factors such as the axonal diameter, density of axons, the intermodal spacing of the myelin, and the degree of myelination itself ^67^. Together with this, mathematicians’ high degree of automaticity in the processing of complex arithmetic formulas seems to be supported by stronger streamline density of the AF/SLF pathway (Fig. 3a). It is interesting to note that the direct pathway of the AF/SLF connecting temporal cortex and Broca’s area plays an eminent role in language for the processing of complex sentence structures^68,69^. Developmental studies comparing the automaticity of processing complex language structures in adults and children reveal that adults, but not 7-year-old children, possess a fully developed direct segment of the AF/SLF pathway, suggesting that the structural maturation of dorsal tract targeting Broca’s area might be the prerequisite for the automatic process of complex structures in language^68^. A recent study also indicated that children with a more mature AF are more accurate and faster in processing complex syntax compared to those who have less maturation of AF^69^. In line with these previous studies, our result also showed that mathematicians who had high level of automaticity (denoted by low CV_RT_) showed a significant correlation with the streamline density of the indirect segment of the AF/SLF. To sum up, we render new evidence that the streamline density of the AF/SLF is related to the degree of automaticity in the processing of complex arithmetic formulas in mathematics.

### Streamline density of the subcortical pathways related to controlled processing in mathematics

In the present study, the streamline density in a cluster within the left thalamus increased as the level of automaticity decreased (denoted as increasing CV_RT_, Fig. 3b). Here, we suggest that cortico-thalamic connections, specifically fibers reaching the thalamus, actively accommodate the performance of demanding and less automatic processes. Previous studies discussed the role of thalamus from the perspective of functional specificity focusing on its activation in association with less automated processing. For example, attention demanding operations revealed thalamic activation with increasing task difficulty^70–72^. The interaction between high order association cortices with the thalamus is necessary for attention demanding tasks where people need to focus on a specific target among multiple distractors^73,74^. Fiber tracts starting from dorsolateral PFC to the thalamus showed increased modulation of the pathway in accordance with increased attentional effort^75,76^. Processing structures composed of random visual symbols or linguistic stimuli in the second language also revealed the activations of subcortical areas including caudate nucleus and thalamus, particularly when the level of processing was demanding^55^. Putting all these studies together, cortico-thalamic pathways may be modulated by the increased cognitive load that coincides with less automaticity^77–79^, resulting in the strong streamline density as the level of automaticity decreased in the present study.

It should be noted that our cortico-thalamic connections are inter-hemispheric, not intra-hemispheric. Such an inter-hemispheric connection is a rather unusual finding, given that most studies quantifying thalamic connectivity exclusively focus on unilateral fiber tracts. However, there are a few tracer studies that report contralateral thalamo-cortical connections in rodents^80,81^ and primates^82,83^, even though their functional importance has not been fully understood. For humans, the potential role of inter-hemispheric thalamo-cortical connectivity for cognition cannot be easily determined. As a rare example, Philip *et al*.^84^, using a connectivity-based parcellation, demonstrated that right thalamic volume, derived from connectivity with the left precentral regions, correlated significantly with performance in a motor coordination task. Given that our inter-hemispheric connection should be interpreted with caution, future work is needed to delineate the structural characterization and functional role of contralateral thalamo-cortical connectivity in humans.

### Methodological considerations of probabilistic tractography and streamline density

Methodological advantages and disadvantages inherent to the use of probabilistic tractography and streamline density as a measure of structural connectivity are worth noting here. Even though this method has been used repeatedly for measuring connectivity strength of white matter pathways, one should consider its limitations. For instance, the quality of tractography is influenced by various factors including subject motion, physiological noise or hardware limitations that determine spatial resolution^85^. Tracer studies in monkeys revealed that probabilistic tractography reliably shows prominent pathways^86,87^, while being prone to identify false positive connections^88^. These problems are caused by the lack of a gold standard for validation of tractography. However, even with these critical problems, streamline density has been one of the key methods in assessing white matter structures and their supporting roles in cognitive functions^89^. Unlike single fiber models, tractography based on probabilistic sampling from the distributions of voxel-wise principal diffusion directions allows for higher sensitivity to non-dominant fiber directions. This is a fundamental feature when tracking in areas with complex white matter configurations such as crossing fibers^90^. Tractography based on multi-fiber models has provided valuable information on the role of white matter structures and their changes in various research fields such as cognitive functions (e.g. attention^91^, musical syntax processing^92^, goal directed action control^93^) and disease (e.g. autism^94,95^, Parkinson disease^96^, stuttering^97,98^). Therefore, by using multi-fiber models, we could successfully uncover expertise-related changes in white matter tracts and the correlation of streamline densities of the fibers with the degree of automaticity in different functional domains.

## Conclusion

We provide first *in vivo* evidence for structural connectivity of cortico-cortical and cortico-thalamic pathways reflecting automaticity in the processing of complex arithmetic formulas. These insights were derived from an analysis of white matter structural integrity across a group of experts and non-experts in mathematics. Importantly, seed ROIs for tracking were selected based on fMRI evidence from previous studies allowing a functional allocation of identified tracts. We suggest that high levels of automaticity in mathematics are reflected in the connectivity profile of the left PrCG to temporal brain areas via the left AF/SLF. Concurrently, low levels of automaticity are associated with higher structural integrity of cortico-thalamic connection. In this way, we shed light on the structural connectivity dependent on behavioral characteristics denoting the individual level of expertise.

Beyond our streamline density analysis, several studies have been conducted in terms of expertise or training investigating gray matter volume^9–12,99^, cortical thickness^14,15,21,25^, or white matter changes^11,16,17,100^. Collectively, these insights highlight the importance of comprehensive measures to gain a better understanding of the neural basis underlying experts’ talents. Mastering a cognitive ability to the level of experts modulates the brains’ white matter architecture. Here we have demonstrated this for the higher cognitive function of mathematics. Future research is needed to show whether this holds for other cognitive domains as well, potentially reflecting a more general principle of brain re-organization supporting expertise.

## Methods

### Participants

Participants were identical to those from a previous study^3^. Twenty-two participants with high levels of expertise in mathematics were recruited based on their occupation (i.e. mathematicians or mathematics teachers), while low-expertise participants (n = 22) were not involved in professional mathematics in their daily lives. The different levels of mathematical expertise of both groups were assessed via a standardized mathematics test (Mathematik-Test: Grundkenntnisse für Ausbildung und Beruf^101^). General intelligence (the Berlin Intelligence Structure Test^102^) and verbal working memory span (the German version of the Wechsler subtest^103^) were assessed across all the participants. Details, and demographic and cognitive profiles of the participants are provided in Table 2 which indicates that cognitive profile of the two groups differed only in mathematics. All the participants gave written, informed consent to participate in the study. The Research Ethics Committee of the University of Leipzig approved the study in accordance with the Declaration of Helsinki.

**Table 2:** Demographic and cognitive profile of mathematicians and non-mathematicians.

	Experts	Non-experts	Statistics
Age	29.78 (7.53)	29.83 (6.76)	*p* = 0.139
Gender: M/F	16/6	14/8	*p* = 0.373
Handedness: LQ	90.26 (9.55)	93.35 (12.45)	*p* = 0.351
Years of education	18.8 (3.2)	17.45 (2.85)	*p* = 0.139
Mathematics test	70.39 (7.05)	42.96 (9.58)	*p* < 0.001
Intelligence test	116.39 (13.81)	126.87 (16.72)	*p* = 0.084
WM (forward)	9.78 (2.15)	9.43 (2.1)	*p* = 0.582
WM (backward)	8.98 (2.4)	8 (2.04)	*p* = 0.106

Values depict mean (SD); statistics were obtained from independent t-tests except for gender (Pearson’s chi-square test). LQ, laterality quotient^116^; WM, working memory.

### Assessment of mathematical automaticity

The degree of automaticity in mathematics was measured by the coefficient of variation in reaction time (CV_RT_) obtained from the participants’ performance at solving a series of 150 algebraic expressions in the previous fMRI study^3^. CV_RT_ is defined as the standard deviation of reaction time (SD_RT_) divided by the mean reaction time (Mean_RT_). CV_RT_ has been used to distinguish between speed-up (improvement without increased automaticity) and restructuring (improvement with increased automaticity), providing an index of processing efficiency associated with automaticity^104^. Restructuring appears in the process of automatization, which qualitatively changes the underlying processes such as reorganization, routinization, or bypassing of serial execution of sub-processes in the course of performance development^105^. In speed-up, CV_RT_ is reduced with an upper limit proportional to the change in RT itself. On the contrary, in restructuring, CV_RT_ is reduced more than proportional to the RT due to variables involved in controlled processes being discarded (i.e., self-monitoring, error correction, or resolving signal-to-noise processing problems). Therefore, CV_RT_ decreases in the case of automatization while remaining unchanged in the case of speed-up^104–106^. For statistical analysis, we conducted Wilcoxon ranked-sum test because we detected non-normality of the data after running Shapiro-Wilk normality test.

### Diffusion MRI data acquisition

Diffusion-weighted MRI data were acquired on a whole-body 3 Tesla Tim Trio MRI scanner (Siemens Healthcare, Erlangen, Germany) equipped with an 32-channel phased-array head coil using a twice-refocused spin echo echo-planar-imaging sequence (TR = 12900 ms; TE = 100 ms; 128 × 128 image matrix, FOV = 220 × 220 mm; 88 axial slices; resolution: 1.72 × 1.72 × 1.7 mm³; 60 uniformly distributed diffusion-encoding gradient directions with a b-value of 1000 s/mm²; GRAPPA 2)^107,108^. Additionally, seven datasets with no diffusion weighting (b0) were acquired initially and interleaved after each block of 10 diffusion-weighted images. An anatomical high-resolution T1-weighted scan was acquired on the same scanner, using a 3D magnetization prepared rapid gradient echo (MPRAGE) sequence^109^ with selective water excitation and linear phase encoding (TR = 2300 ms; TE = 2.96 ms; 256 × 240 image matrix; FOV = 256 × 240 mm; 176 axial slices; resolution: 1 × 1 × 1 mm³).

### Diffusion MRI preprocessing

Diffusion MRI data were screened for motion induced signal dropouts with a semi-automatic method^110^. Additionally, the data were visually inspected for artifacts^111,112^. Following this procedure, we had to exclude two mathematicians and four non-mathematicians from the final analysis due to excessive head motion or unavailability of suitable diffusion-weighted scans. Thus, dMRI data from 38 participants was used for the final analysis. Preprocessing of dMRI data was performed using FSL v5.0^113^. For each participant, diffusion data was divided into volumes with and without diffusion weighting. Separate averages for each subset were computed. These averages were rigidly aligned to the T1-weighted image previously aligned to Montreal Neurological Institute (MNI) standard space and interpolated to 1 mm voxel size. For motion correction, volumes with and without diffusion weighting were rigidly aligned to their respective average in MNI space. To preserve high data quality, all transformations necessary for motion correction and registration to the individual T1 anatomy in MNI space were combined and applied in a single step of interpolation. The fiber orientation distribution for each voxel was determined using bedpostX^90^. Additionally, transformation matrices for affine registration of each participant’s T1 data to the standard MNI152_T1_1 mm_brain.nii.gz image as provided in FSL were computed using FSL’s flirt for later registration of individual tractograms to a common standard space.

### Structural connectivity analysis

In order to obtain white matter pathways associated with behavioral variation, we selected areas from a previous study^3^ where both groups (mathematicians and non-mathematicians) showed common activations in the processing of complex arithmetic formulas (Fig. 1a compared to Fig. 1b): left insula, left PrCG, left SPL and bilateral mPMC encompassing ACC. These ROIs were extracted in volume space using the MarsBaR toolbox in SPM^114^. Subsequently, each seed was resampled to 1mm resolution and affinely aligned with the individual participant’s T1 data in MNI space using FSL’s flirt. The necessary transformation matrix for this registration was the inverse of a previously computed transformation from the individual participant’s T1 MNI space to the standard space of the seeds.

Tractography was performed using probtrackx2^90^. For each seed, 5000 streamlines were initiated from each voxel on the grey matter-white matter interface within the seed region; using a curvature threshold of 0.2 and step length of 0.5 mm. Tracking was restricted to the white matter only. The corresponding white matter mask was generated by fitting the diffusion tensor (FSL dtifit) and thresholding the resulting fractional anisotropy (FA) map at 0.2. The resulting streamline density maps were first logarithmized and normalized by dividing each voxel by the logarithm of the maximal possible number of streamlines produced. Subsequently, each preprocessed tractogram was affinely aligned to the MNI152_T1_1mm_brain.nii.gz image as provided in FSL, based on the transformations from T1 data to this image previously computed. Further, all MNI-aligned tractograms were averaged, that is, they were summed up and divided by the total number of available tractograms (i.e. 38). Finally, these averages were thresholded at a value of 0.2 to obtain masks for statistical analysis (see Fig. 2). Statistical analyses were performed by running non-parametric regressions (FSL randomize^115^) with 10000 Monte Carlo simulations. To test for respective linear associations, we set up general linear models with CV_RT_ as a single regressor. Thus, we examined which regions of the logarithmized and normalized individual streamline density maps in MNI152 space correlated with CV_RT_ scores within the regions defined by the average tract mask in a voxel-wise fashion. Reported clusters for individual tracts were significant at the voxel and cluster levels of p < 0.001 and p < 0.05 respectively, Bonferroni corrected for the number of seed regions.

### Data policy

Data in an anonymized form (in accordance to the ethics agreement) and scripts used in data analysis are available on request.

## Supplementary information

Supplementary Information
