# Supplementary material for: Mathematical expertise modulates the architecture of dorsal and cortico-thalamic white matter tracts
Source: Sci Rep. 2019 May 2;9:6825. doi: 10.1038/s41598-019-43400-6 (PMC6497695; doi:10.1038/s41598-019-43400-6)
Supplement: Supplementary file 1 — Supplementary Information [file 41598_2019_43400_MOESM1_ESM.docx]

**Supplementary information**

**Mathematical expertise modulates the architecture of dorsal and cortico-thalamic white matter tracts**

Hyeon-Ae Jeon^1,2,+,*^, Ulrike Kuhl^3,+^, Angela D. Friederici^3^

^1^Department of Brain and Cognitive Sciences, Daegu Gyeongbuk Institute of Science and Technology (DGIST), Daegu 42988, Korea

^2^Partner Group of the Max Planck Institute for Human Cognitive and Brain Sciences at the

Department for Brain and Cognitive Sciences, DGIST, Daegu 42988, Korea

^3^Department of Neuropsychology, Max Planck Institute for Human Cognitive and Brain Sciences, Leipzig, 04103, Germany

**Supplementary Figure S1.** **Separate correlations for mathematicians and non-mathematicians.**


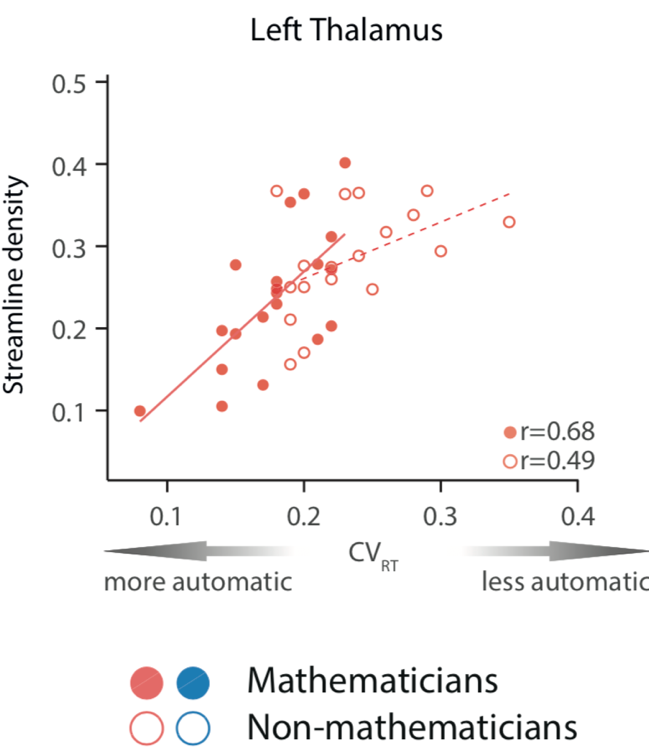


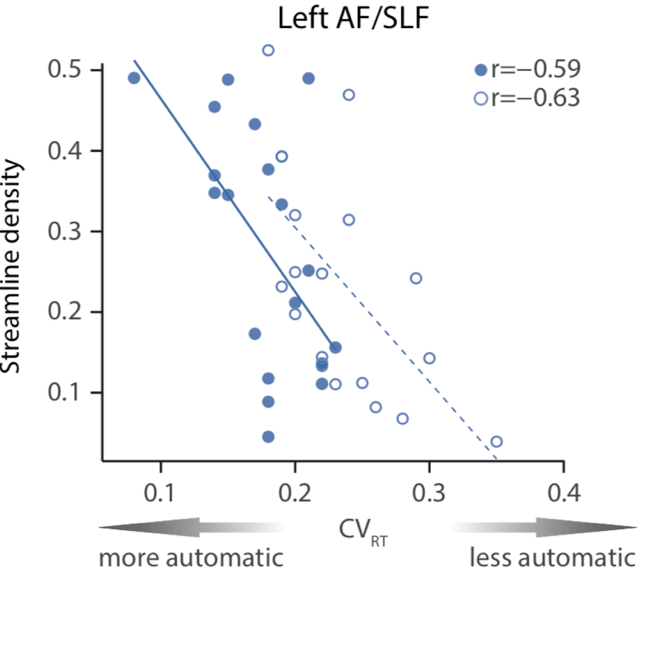


(a) For streamline density within the left AF/SFL cluster, a significant negative correlation was found with CV_RT_ scores for mathematicians (r = -0.59, p = 0.0063) and non-mathematicians (r = -0.63, p = 0.0053), respectively. (b) The positive association between streamline density within the left thalamus cluster and CV_RT_ scores were also significant for both groups (mathematicians: r = 0.68, p = 0.0009; non-mathematicians: r = 0.49, p = 0.0391). An additional statistical comparison of both sets of correlations using Fisher’s z revealed no significant differences between the strength of associations between both groups (AF/SLF cluster: z = -0.1800, p = 0.8572; left Thalamus cluster: z = -0.8273, p = 0.4081).
